# Antimicrobial susceptibility and integrons detection among extended-spectrum β-lactamase producing Enterobacteriaceae isolates in patients with urinary tract infection

**DOI:** 10.7717/peerj.15429

**Published:** 2023-06-02

**Authors:** Karzan Taha Abubaker, Khanda Abdulateef Anwar

**Affiliations:** 1 Microbiology Department/Shar Teaching Hospital, Sulaimania Directorate of Health, Sulaimani, Sulaimani, Iraq; 2 Microbiology Department/College of Medicine/University of Sulaimani, Sulaimani, Sulaimani, Iraq

**Keywords:** Integrons, β-lactamase, Plasmid, Urinary tract infection, Cross-sectional study

## Abstract

**Background:**

Integrons are bacterial mobile genetic components responsible for mediating the antibiotic resistance process by carrying and spreading antimicrobial resistance genes among bacteria through horizontal gene transfer.

**Objectives:**

This cross-sectional hospital-based study aimed to find the prevalence of antibiotic resistance patterns and to detect integrons classes (I, II, and III) among bacterial isolates in patients with urinary tract infections (UTI) in Sulaimani, Iraq.

**Patients and Methods:**

Mid-stream urine samples (no. = 400) were collected from patients with UTI at three different Hospitals from Sulaimani, Iraq, between September 2021 to January 2022. Urine samples were cultured on various agar media, and grown bacteria were isolated. Antibiotic susceptibility test (AST) and an extended-spectrum β-lactamase (ESBL) screen were done for isolated bacteria. Then, integrons classes were screened using conventional PCR with gene sequencing and uploaded to the National Center for Biotechnology Information (NCBI).

**Results:**

The frequency rate of *Enterobacteriaceae* was 67.03% among positive urine cultures. *E. coli* (no. = 86) and *Klebsiella pneumoniae* (no. = 32) isolates were identified. The most sensitive antibiotics were the carbapenem group (85.3%) and nitrofurantoin (NFN) (64.2%), while the most resistant antibiotics were nalidixic acid (NA) and 3^rd^ generation cephalosporin. The occurrence rate of ESBL was 56.6% with a predominance of class I integron (54.2%), then class II (15.8%) and no positive record for class III integron were observed.

**Conclusion:**

Most bacterial isolates from patients with UTI produced class I and II integrons genes with favourable ESBL properties.

## Introduction

The frequency of urinary tract infection (UTI) by extended-spectrum β-lactamase (ESBL)-producer *Enterobacteriaceae* has recently become augmented, especially *E. coli* (75–90%). Unfortunately, early experimental treatment is regularly unsuccessful for these tough isolates, resulting in lengthy hospitalization and death (Lee et al., 2018).

Increased prevalence of ESBL-producing *E. coli* is a common task for clinical physicians in patients with UTI as ESBL can degrade the β-lactam ring of most of the penicillins, cephalosporins and aztreonam. Additionally, some ESBL plasmids harbour genes that bring them resistance to aminoglycosides, sulfonamides, and fluoroquinolones. Thus, therapeutic choices for UTI due to ESBL *E. coli* are limited and restricted (Zhu et al., 2019).

Integrons are genes that acquire exogenous medication resistance properties and let their manifestation in some bacterial species. It is essential in the horizontal spread of bacterial antibiotic resistance through the integron-gene cassette system (Escudero, Loot & Mazel, 2018). The action of integrase causes bacteria to detention and direct foreign genes that produce drug resistance, leading to both pan- and multidrug resistance (Faghri et al., 2018; Ghaly et al., 2021).

The proliferation of strains expressing ESBL enzymes has resulted in a rise in antibiotic resistance among Gram-negative bacteria through several mechanisms, including integrons. The coding genes of ESBL are found on bacterial chromosomes, which can be inherited or acquired through plasmids, allowing them to migrate between bacterial populations (Benz et al., 2021; Kaur et al., 2013). Based on the arrangement of their genes, integrons are categorized into three classes. However, class I is more commonly identified. Integrons can localize within conjugative plasmids or transposons that harbour integrase enzymes. Most class I integrons are linked to cassettes of capture and can capture more resistant genes, such as ESBL, that code for multidrug-resistant properties genes from a vast collection of resistance genes that give antibiotic resistance (Deng et al., 2015).

Thus, we aimed to find the prevalence of antibiotic resistance and determine integrons classes of isolated species of *Enterobacteriaceae* from patients with UTI in Sulaimani, Iraq.

## Materials and Methods

### Subjects

This cross-sectional study involved 400 patients (inpatients and outpatients) with the signs and symptoms of UTI who were admitted to Shar Teaching Hospital, Sulaimani Teaching hospital, and Anwar Shexa Medical City Hospital Sulaimaniyah, Iraq, from September 2021 to January 2022.

### Inclusion criteria

Patients with confirmed UTI, regardless of gender, ethnicity, and nationality.

### Exclusion criteria

Pediatric age group (less than 7 years old) and pregnant ladies.

### Questionnaire

A well-designed, self-prepared questionnaire was used to collect the patient’s sociodemographic data such as age, gender, catheterization condition, hospitalization type, UTI recurrence and type, with their medical history and comorbidities.

### Bacterial isolation

The early morning midstream urine specimens were requested to be inoculated into different culture media and then incubated for 24–48 h at 37 °C in aerobic situations (Karah et al., 2020). VITEK 2 compact (BioMerieux, Marcy-l′Étoile, France) was used to identify isolated *Enterobacteriaceae* that produces a pink color colony with green metallic sheen property on Eosin Methylene Blue (EMB) agar according to the manufacturer’s recommendation. The bacterial stock was prepared in an Eppendorf tube and stored in a deep freezer at −40 °C for further analysis (Sprouffske, Aguilar-Rodríguez & Wagner, 2016).

### Antibiotic susceptibility test (AST)

Using commonly available antibiotic discs, all the bacterial isolates were tested using the Kirby-Bauer disc diffusion technique according to what was fixed by Clinical, & Institute, Laboratory Standards (2020). The commonly used discs were amoxiclav (AMC) (20 μg amoxicillin [AMOX] + 10 μg clavulanic acid [CLA]), ceftazidime (CAZ; 30 μg), ceftriaxone (CRO, 30 μg), cefotaxime (CTX; 30 μg), cefixime (CFM; 5.0 μg), cefepime (CPM; 30 μg), ciprofloxacin (CIP; 5.0 μg), trimethoprim-sulfamethoxazole (SXT; 5.0 μg), nalidixic acid (NA; 10 μg), nitrofurantoin (NFN; 10 μg), gentamicin (GEN; 10 μg), imipenem (IPM; 10 μg), and meropenem (MEM; 10 μg).

### ESBL screen test

A double-disk synergy test (DDST) was used to screen ESBL with the positive keyhole phenomenon (Kaur et al., 2013) and confirmed by two combined discs (CD) test methods such as cefotaxime-clavulanic acid disc and cefepime-clavulanic acid disc (Anoar, Ali & Omer, 2014; Teklu, 2019) and using AST GN74 cards (BioMerieux, Marcy-l′Étoile, France).

*Klebsiella pneumoniae* (ATCC 700603) was utilized as a positive control for ESBL, while *E. coli* (ATCC 25922) was used as a negative control.

### Molecular detection of integron classes

Genomic DNA was extracted using the boiling method (colony PCR). Briefly, 2–3 pure isolated colonies from an overnight cultured bacterial growth in blood agar were taken and suspended in 200 μL of sterile distilled water, boiled at 95 °C for 10 min in the water bath. Then, the suspensions were centrifuged at 13,000 rpm for 10 min to remove cellular debris. The supernatant was used as the template for amplification and stored in an Eppendorf tube at −40 °C until use (Lan et al., 2019).

All isolates were screened for classes I, II, and III integrons using conventional polymerase chain reaction (PCR) according to specific conditions and primers applied previously (Kargar et al., 2014). Briefly, the PCR reaction was done based on the manufacturer’s instructions using the Add Star Taq master mix PCR kit (Add Bio, Gyeongbuk, Republic of Korea), which contains 20 mM Tris-HCL (pH 8.8), 100 mM KCL, 0.2% Triton X100, 4.0 MgCl_2_, a protein stabilizer, loading dye and 0.5 mM each of dATP, dCTP, dGTP, and dTTP. Then, PCR reaction was accomplished to 20 µL by DEPC-H_2_O (3.0 µL), 1.0 µL of 10 pmol forward/reverse primers, and 5.0 µL of a DNA sample. Next, the thermocycler (ESCO Thermocycler; ESCO, Singapore) was set for denaturation, annealing, and a final extension phase. Table 1 illustrates the PCR condition of primers and their sequences.

**Table 1:** Primer sequences and PCR application condition for integron I–III.

Variable	Gene		
	Integron I	Integron II	Integron III
Forward Direction	TCTCGGGTAACATCAAGG	CACGGATATGCGACAAAAAGG	AGTGGGTGGCGAATGAGTG
Reverse Direction	AGGAGATCCGAAGACCTC	TGTAGCAAACGAGTGACGAAATG	TGTTCTTGTATCGGCAGGTG
Size (bp)	294	740	600
Initial Denaturation	95 °C/5 min	95 °C/5 min	95 °C/5 min
Denaturation	95 °C/30 s	95 °C/30 s	95 °C/30 s
Annealing	58 °C/30 s	60 °C/30 s	60 °C/30 s
Extension	72 °C/30 s	72 °C/30 s	72 °C/30 s
Final extension	72 °C/5 min	72 °C/5 min	72 °C/5 min

The amplicons were analyzed by electrophoresis on 1.0% w/v agarose gel in TBE buffer for 60 min and a voltage of 90. Then, the results were assessed under UV light on the UV transilluminator.

### Sequencing of PCR products

The purified PCR products of four samples (two for integron class I and two for integron class II) were sequenced by the Macrogen Genome Center (Seoul, Republic of Korea). Then, the sequences were analyzed using Chromas Technelysium with online BLAST software (http://www.ncbi.nlm.nih.gov/BLAST/).

### Ethical approval

All measures accomplished in this study are based on the ethical standards of the National Research Committee and the Helsinki declaration, 1964 and its later amendments or comparable ethical standards. Simultaneously, written informed consent was obtained from the patients to publish their data. Therefore, the Scientific and Ethical Committee of the Sulaimani Directorate of Health and the Ethics Committee of the College of Medicine, University of Sulaimani, Iraq, approved the research protocol (No. 170-CoM-UoS on September 14, 2021).

### Statistical analysis

Statistical Package performed data analysis for Social Science (SPSS Inc., Chicago, IL, USA, version 26). The Chi-square test was used for the association between variables. Descriptive statistics were presented as mean ± standard deviation (SD) and frequency/percentages for categorical variables. The significance level was defined at p ≤ 0.05, while highly significant was set at *p* ≤ 0.001.

## Results

### Sociodemographic characteristics of the patients

The participant’s ages in this study ranged from 7–84 years, with a mean of 45.5 years. The primary age groups that attended hospitals with UTI symptoms were elderly patients (>60 years), and females were more affected (69%) than males. Among the studied patients, 110 were hospitalized, and 17% were catheterized for different conditions. On the other hand, most patients had UTI for the first time (69.5%), mainly due to cystitis (56.3%). In comparison, the most prevalent comorbidities among patients were diabetes (35%), and 42% were without medical history. Consequently, to examine if the positive culture rate was associated with any patient socio-demographics, catheterization status, hospitalization condition, UTI status/type, and medical history of the patients were investigated, and a highly significant (*p* < 0.001) correlation was found between the variables and the rate of positive cultured samples (Table 2).

**Table 2:** Socio-demographic characteristics of the patients with UTI according to positive and negative urine culture.

Variable		Patient with positive culture (No., %)	Patient with negative culture (No., %)	Total (No., %)	*p*-value
Age (Year)	<15	9 (39.1)	14 (60.9)	23 (5.75)	0.99
	15–30	17 (42.5)	23 (57.5)	40 (10.0)	
	31–45	38 (42.2)	52 (57.8)	90 (22.5)	
	46–60	43 (43.0)	57 (57.0)	100 (25.0)	
	>60	64 (43.5)	83 (56.5)	147 (36.75)	
Gender	Male	45 (42.7)	79 (57.3)	124 (31.0)	0.08
	Female	126 (42.8)	150 (57.2)	276 (69.0)	
Catheterization	Catheterized	60 (88.2)	8 (11.8)	68 (17.0)	<0.001*
	Non-catheterized	111 (33.4)	221 (66.6)	332 (83.0)	
Hospitalization	Inpatients	90 (81.8)	20 (18.2)	110 (27.5)	<0.001*
	Outpatients	81 (27.9)	209 (72.1)	290 (72.5)	
Recurrence	First time UTI	76 (27.3)	202 (72.7)	278 (69.5)	<0.001*
	Repeated UTI	95 (77.9)	27 (22.1)	122 (30.5)	
Type of UTI	Cystitis	78 (34.7)	147 (65.3)	225 (56.3)	<0.001*
	Pyelonephritis	76 (60.8)	49 (39.2)	125 (31.3)	
	Urethritis	11 (25.6)	32 (74.4)	43 (10.7)	
	Prostatitis	6.0 (85.7)	1.0 (14.3)	7 (1.7)	
Medical history	Diabetic	44 (31.4)	96 (68.6)	140 (35.0)	<0.001*
	Hypertension	42 (60.0)	28 (40.0)	70 (17.5)	
	Chronic kidney disease	21 (95.5)	1.0 (4.5)	22 (5.5)	
	None	64 (38.1)	104 (61.9)	168 (42.0)	
Total		171 (42.7)	229 (57.3)	400	

**Notes:**

* Significant difference using Chi-square test.

UTI, urinary tract infection.

### Patient’s urine culture results

**Figure 1:**
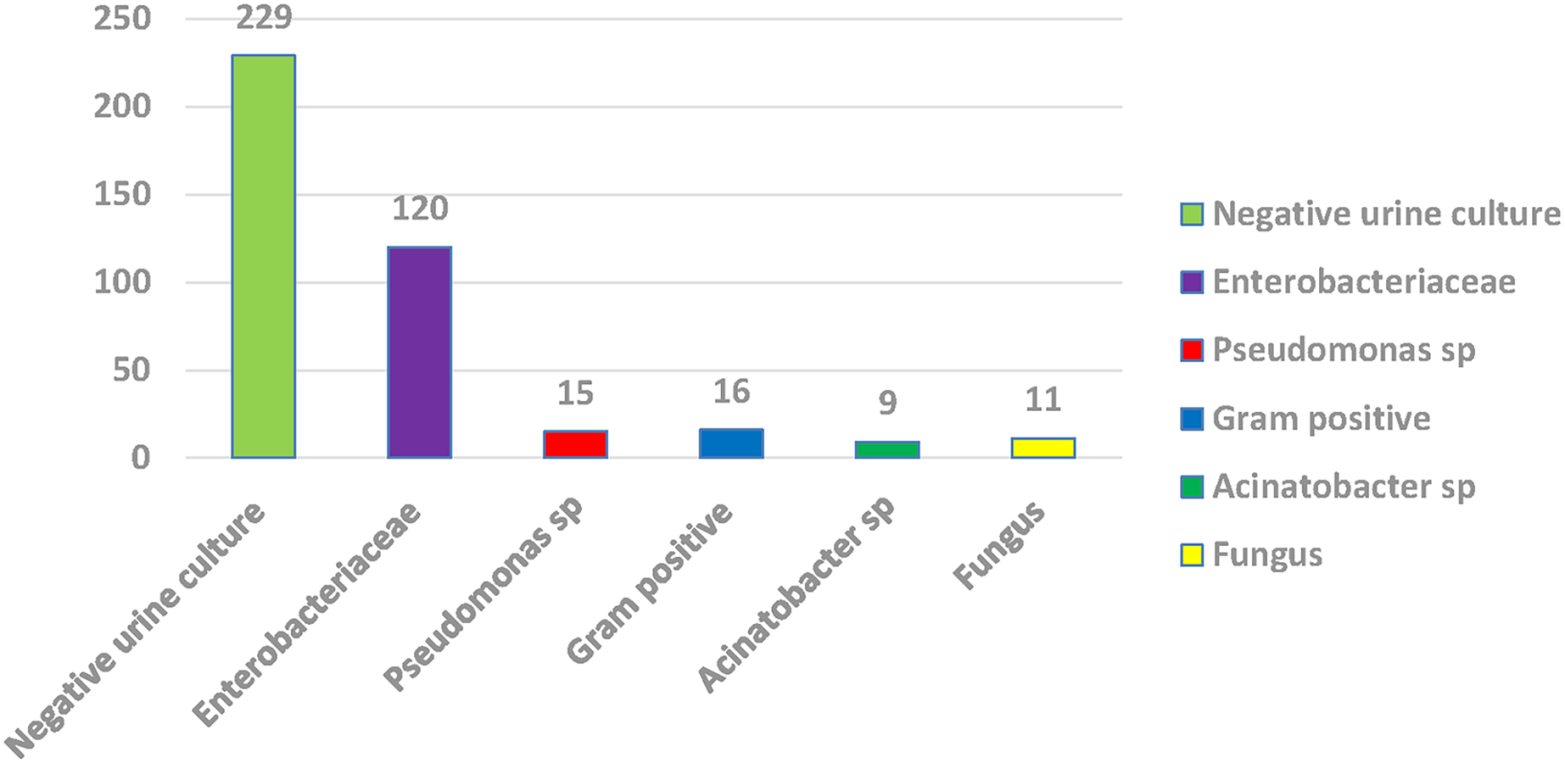
Percentage of positive and negative urine culture with isolated bacteria.

Among 400 urine samples, 171 (42.75%) were positive for urine culture, including *Enterobacteriaceae* (120 samples), followed by Gram-positive bacteria (no. = 16) and non-fermenter Gram-positive bacteria (no. = 16), then Pseudomonas species (no. = 15), fungi (no. = 11) with Acinetobacter species (no. = 9). Among *Enterobacteriaceae*, 86 (71.7%) isolates were *E. coli*, 32 (26.7%) were *Klebsiella pneumoniae*, and 2 (1.7%) were *Proteus mirabilis* (Fig. 1).

### Bacterial AST outcomes

The most resistant antibiotics in this study for all isolated *Enterobacteriaceae* species (*E. coli*, *Klebsiella pneumoniae*, and *Proteus mirabilis*) were 3^rd^ generation cephalosporins (CTR, CAZ, CTX), followed by NA (78.35%), SXT (70%), then CIP (55.8%), and CPM (46.7%). At the same time, the most sensitive antibiotic for all three isolates was IPM (85.8%), followed by MEM (67.5%) and NFN (64.2%) (Table 3).

**Table 3:** Antibiotic profile of isolated *Enterobacteriaceae*.

Antibiotic disc	Susceptibility	Bacterial name			Total (No., %)
		*E. coli* (No., %)	*Klebsiella pneumoniae* (No., %)	*Proteus mirabilis* (No., %)	
**Amoxiclav** **(AMC)**	**R**	38 (44.2)	15 (46.9)	1 (50.0)	54 (45.0)
	**I**	5 (5.80)	2 (6.3)	0 (00.0)	7 (5.8)
	**S**	43 (50.0)	15 (46.9)	1 (50.0)	59 (49.2)
**Ceftazidime** **(CAZ)**	**R**	64 (74.4)	23 (71.9)	2 (100)	89 (74.2)
	**I**	2 (2.3)	2 (6.3)	0 (00.0)	4 (3.3)
	**S**	20 (23.3)	7 (21.9)	0 (00.0)	27 (22.5)
**Cefotaxime** **(CTX)**	**R**	62 (72.1)	21 (65.6)	2 (100)	85 (70.8)
	**I**	3 (3.5)	0 (00.0)	0 (00.0)	3 (2.5)
	**S**	21 (24.4)	11 (34.4)	0 (00.0)	32 (26.7)
**Ceftriaxone** **(CTR)**	**R**	65 (75.6)	23 (71.9)	2 (100)	90 (75.0)
	**I**	2 (2.3)	2 (6.3)	0 (00.0)	4 (3.3)
	**S**	19 (22.1)	7 (21.9)	0 (00.0)	26 (21.7)
**Cefepime** **(CPM)**	**R**	43 (50.0)	13 (40.6)	0 (00.0)	56 (46.7)
	**I**	1 (1.2)	0 (00.0)	0 (00.0)	1 (0.8)
	**S**	42 (48.8)	19 (59.4)	2 (100)	63 (52.5)
**Imipenem** **(IPM)**	**R**	14 (16.3)	2 (6.3)	0 (00.0)	16 (13.3)
	**I**	1 (1.2)	0 (00.0)	0 (00.0)	1 (0.8)
	**S**	71 (82.6)	30 (93.8)	2 (100)	103 (85.8)
**Meropenem** **(MEM)**	**R**	25 (29.1)	6 (18.8)	2 (100)	33 (27.5)
	**I**	5 (5.8)	1 (3.1)	0 (00.0)	6 (5.0)
	**S**	56 (65.1)	25 (78.1)	0 (00.0)	81 (67.5)
**Nitrofurantoin (NFN)**	**R**	30 (34.9)	10 (31.3)	2 (100)	42(35.0)
	**I**	0 (00.0)	1 (3.1)	0 (00.0)	1 (0.8)
	**S**	56 (65.1)	21 (65.6)	0 (00.0)	77 (64.2)
**Nalidixic acid** **(NA)**	**R**	67 (77.9)	25 (78.1)	2 (100)	94 (78.3)
	**I**	4 (4.7)	0 (00.0)	0 (00.0)	4 (3.3)
	**S**	15 (17.4)	7 (21.9)	0 (00.0)	22 (18.3)
**Gentamicin** **(GNM)**	**R**	39 (45.3)	12 (37.5)	2 (100)	53 (44.2)
	**I**	5 (5.8)	4 (12.5)	0 (00.0)	9 (7.5)
	**S**	42 (48.8)	16 (50.0)	0 (00.0)	58 (48.3)
**Ciprofloxacin** **(CIP)**	**R**	51 (59.3)	14 (43.8)	2 (100)	67 (55.8)
	**I**	6 (7.0)	3 (9.4)	0 (00.0)	9 (7.5)
	**S**	29 (33.7)	15 (46.9)	0 (00.0)	44 (36.7)
**Trimethoprim-Sulfamethoxazole (SXT)**	**R**	62 (72.1)	20 (62.5)	2 (100)	84 (70.0)
	**I**	8 (9.3)	1 (3.1)	0 (00.0)	9 (7.5)
	**S**	16 (18.6)	11 (34.4)	0 (00.0)	27 (22.5)

**Note:**

R, Resistance; I, Intermediated; S, Sensitive.

### Bacterial isolates ESBL production

**Figure 2:**
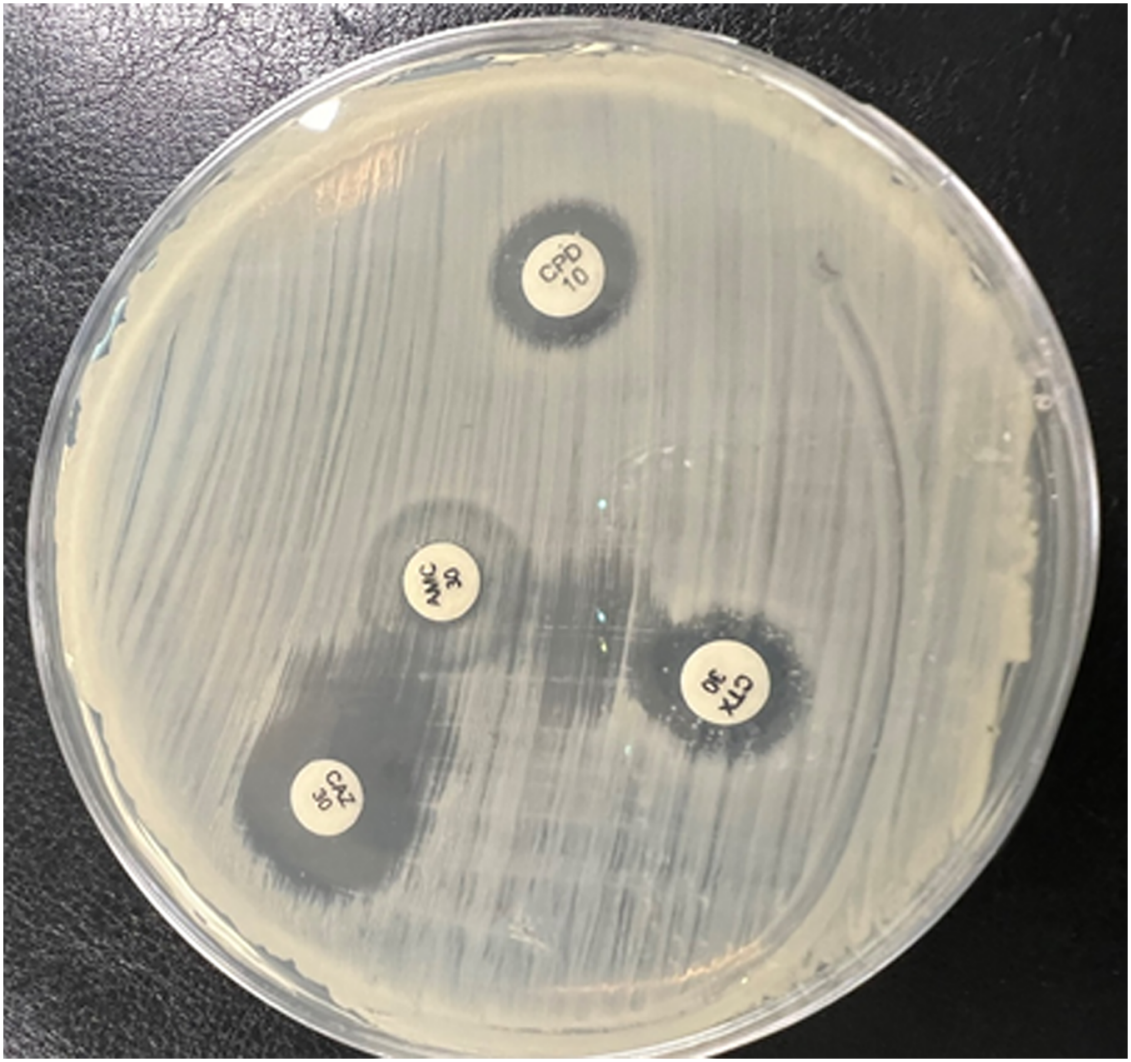
The double-disk synergy test presented extended zones of inhibition by third-generation cephalosporin (CTR, CAZ, CTX) toward the center amoxicillin/clavulanic acid (AMC) disc and formation of the keyhole phenomena.

All isolated *Enterobacteriaceae* bacteria were produced ESBL (*p* > 0.05) using a double disc synergy test (DDST). The zone of inhibition towards the amoxicillin-clavulanate (AMC) disc is shown in Fig. 2.

Then, two phenotypic methods were used to confirm ESBL production. CD1 recorded 56.6% against all species, and the value decreased to 53.3% by automated method (VITEK System). *E. coli* (71.7%) was the highest ESBL producer, followed by *Klebsiella pneumoniae* (26.7%), while *Proteus mirabilis* (1.7%) was the minor producer (Table 4). Regarding the ESBL screen through the antibiotic’s profile, 74.2% of isolated Gram-negative bacteria were resistant to the 3^rd^ generation of cephalosporins (CTR, CAZ, CTX). Out of these isolates, different ranges were found using DDST. Additionally, CD2 was used to confirm fourth-generation cephalosporins (cefepime) resistance among isolated bacteria, which was 15.8% (*p* = 0.108) (Table 5).

**Table 4:** Prevalence of ESBL among *Enterobacteriaceae* by different screening and confirmatory tests.

Bacterial isolates	Screening test (No., %)	DDST (No., %)	Confirmation of ESBL		Total (No., %)
			CD1 (No., %)	VITEK system (No., %)	
*E. coli*	64 (74.4)	51 (59.3)	53 (61.6)	50 (58.1)	86 (71.7)
*Klebsiella pneumoniae*	23 (71.9)	13 (40.6)	13 (40.62)	13 (40.62)	32 (26.7)
*Proteus mirabilis*	2 (100)	2 (100)	2 (100)	1 (50)	2 (1.7)
Total	89 (74.2)	66 (55)	68 (56.6)	64 (53.3)	120 (100)
*p*-value*	0.675	0.084	0.057	0.23	>0.05

**Notes:**

* Chi-square test.

CD1, Combined Disk 1 (Cefotaxime plus Clavulanic acid); DDT, Double Synergy Disk Test; Screen test 1: Resistance to the third generation of cephalosporin.

**Table 5:** Prevalence of ESBL by combined disk test 2 (CD2).

Isolated bacteria	Resistance to cefepime (No., %)	CD2 (No., %)	Total (No., %)	*p*-value*
*E. coli*	43 (50.0)	16 (18.7)	86 (71.7)	0.108
*Klebsiella pneumoniae*	13 (40.6)	2 (6.3)	32 (26.7)	
*Proteus mirabilis*	0 (00.0)	1 (50)	2 (1.7)	
Total	56 (46.6)	19 (15.8)	120 (100.0)	

**Notes:**

* Chi-square test.

CD2, Combined Disk 2.

### The prevalence rate of integrons classes among isolated *Enterobacteriaceae*

**Figure 3:**
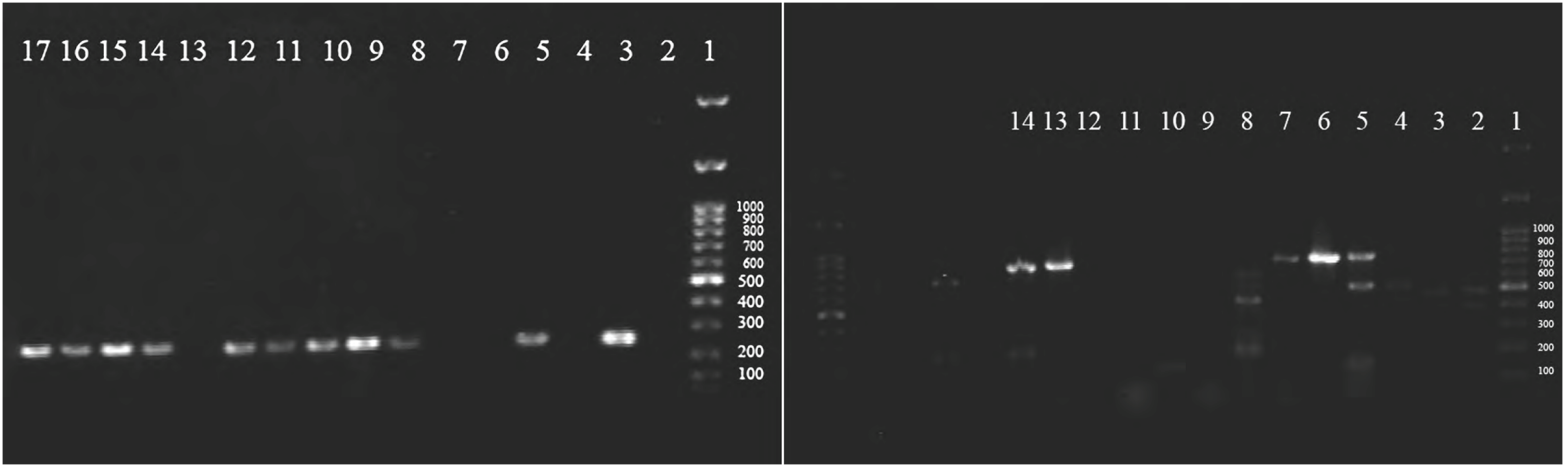
Agarose gel electrophoresis (1%) shows positive class I integron among isolated Enterobacteriaceae. 1: DNA ladder (1Kbp), 3,5,9: positive *Klebsiella pneumoniae*, 10,11,12,14, 15,16,17: positive *E. coli* (left side) and positive c.

All classes of integrons (I, II, III) were screened among isolated *Enterobacteriaceae* by PCR, and clear, joyous bands for classes I and II were observed (Fig. 3). The prevalence rate of class I was 54.2%, while for class II, integrons were 15.8%, and no isolate was positive for class III integrons (Table 6). Class I integron was among the commonest ESBL producers (66.2%) and non-ESBL producers (38.5%) of *Enterobacteriaceae* (*p* = 0.023).

**Table 6:** Isolated integrons in ESBL producer bacteria.

ESBL test	Class I		Class II		Total	*p*-value
	Positive (No., %)	Negative (No., %)	Positive (No., %)	Negative (No., %)	(No., %)	
Positive	45 (66.2)	23 (33.8)	12 (17.6)	56 (82.4)	68 (56.6)	0.023*
Negative	20 (38.5)	32 (61.5)	7 (13.5)	45 (86.5)	52 (43.4)	
Total	65 (54.2)	55 (45.8)	19 (15.8)	101 (84.2)	120 (100)	

**Note:**

* Significant difference using Chi-square test.

### Sequence analysis of integron classes

When sequence data from two samples of class I integron products were aligned using the Chromas Technelysium program, the amplified PCR of class I integron product belongs to one of several resistance genes integrated into *E. coli* and *Klebsiella pneumoniae*. When the forward primer data sequence was uploaded into the BLAST query, the same result of integron class I was obtained. In addition, class II products were aligned using Chromas Technelysium programs, and class II was accepted, Both sequence data were accepted by NCBI provided by GenBank nucleotide sequence accession numbers (ON745429, ON745430, ON745431, and ON745432).

## Discussion

One mechanism of antibiotic resistance is the presence of the mobile genetic elements in *Enterobacteriaceae* named the integron system, which plays a crucial role in the maintenance and spread of antimicrobial resistance among bacteria, especially among Gram-negative bacteria (Pormohammad et al., 2019).

In the current study, among 400 tested urine patients, only 42.7% were culture positive, close to the outcomes of a Bangladeshi study that detected 453 positive urine cultures among 4,000 suspected UTI patients (Nobel et al., 2021). Furthermore, the age range of studied UTI patients was 7–84 years, with a mean of 45.5 years, and most were females (69%). These outcomes are in line with that found by a study in Bangladesh, which reported the UTI patient’s average age was 6–87 years with a mean age of 43.87 ± 22.8, and a higher rate of infected females (77.27%) (Hossain et al., 2020), while another study found that 66.2% of their studies patients with UTI were females with the mean age of 45.50 years (Nobel et al., 2021).

The most resistant antibiotics in this study for isolated *Enterobacteriaceae* species were 3^rd^ generation cephalosporins, followed by NA, SXT, CIP, and CPM. In contrast, the most sensitive antibiotics for isolates were IPM, followed by MEM and NFN. In this regard, the highest rates of bacterial resistance to antibiotics were found to be AMOX (90.0%), NA (78.7%), cephalexin (CPN; 84.1%), CFM (57.5%), CRO (51.2%), and SMX (47.1%) (Hossain et al., 2020).

In this study, 120 bacterial isolates belonging to *the Enterobacteriaceae* family, with *E. coli* at the highest level (71.7%), followed by *Klebsiella pneumoniae* (26.7%), while *Proteus mirabilis* reported the lowest rate (1.7%). These outcomes are not agreed with that found in Iran, in which the most frequent *Enterobacteriaceae* bacteria were; *Klebsiella pneumoniae* 75 (24; 32% ESBL positive), *E. coli* 69 (6; 8.69% ESBL positive) and Enterobacter spp. 5 (5; 100% ESBL positive). (Bagheri-Nesami et al., 2016). On the other hand, *E. coli* was the only uropathogenic bacterial species found in the patient’s urine culture by another group (Nobel et al., 2021).

All isolated *Enterobacteriaceae* bacteria produced ESBL (*p* > 0.05). *E. coli* was the highest ESBL producer (71.7%), followed by *Klebsiella pneumoniae* (26.7%), while *Proteus mirabilis* was the minor producer (1.7%). These outcomes do not agree with that found in Iran, where the Enterobacteriaceae bacteria most frequently ESBL producer *Enterobacteriaceae* bacteria were Enterobacter spp. (100%), followed by *Klebsiella pneumoniae* (32%) and then *E. coli* (8.69%) (Bagheri-Nesami et al., 2016).

Moreover, in this study, different prevalence rates of integron classes I and II were observed among ESBL-positive strains of *Enterobacteriaceae*, especially *E. coli*, which was the commonest one that harbours both classes of integrons I & II. These findings agree with many studies done in Iran (Alkhudhairy et al., 2019; Barzegar et al., 2022; Ebrahim-Saraie et al., 2018; Kangachar & Mojtahedi, 2018). However, class I integron in Nigeria was highly reported in 18 *E. coli* and only one isolate for each *Klebsiella pneumoniae* and *Proteus mirabilis* (Joy et al., 2021). Another study used multiplex PCR assay to detect the *E. coli* integrase gene. It demonstrated that out of 49 bacterial strains, 26 were class I integrons and no case of bacteria harbouring class 2 or class 3 integrons (Chen et al., 2019). These proved the idea and hypothesis that the ESBL enzyme might aid in transferring and expressing integron genes, especially class I integrons (Domingues, da Silva & Nielsen, 2012).

The findings of this study are new in Iraq, and no previous results determined the prevalence rate of integrons in our locality. The high prevalence rate of class I integrons may be due to the integron’s exceptional capacity to capture numerous drug-resistance genes. Various gene cassette arrays encoding different resistance genes that may confer resistance against multiple drugs have emerged (Delarampour et al., 2020).

Class II were recorded to be lower than class I integron in most studies, including this study. The fact that class II integron was translated to truncate integrase proteins because of an early stop codon TAA after the 178^th^ amino acid is considered a defective integron and cannot integrate and cut resistance gene cassette (Lu et al., 2022).

Regarding class III integron, no species were found to be positive, similar to previous studies’ findings (Alkhudhairy et al., 2019; Barzegar et al., 2022; Chen et al., 2019). This could be due to the low rate of class III integron naturally, its molecular characteristics, and its lower role in spreading resistance genes and only present in a few bacterial species (Ghaly et al., 2021; Kargar et al., 2014). Integron genes were also screened among ESBL-negative *Enterobacteriaceae*, and it was found that many species carried class I and II genes. However, the prevalence rate was lower than ESBL producers. The same results were reported in other studies in Gezza and India (Bhattacharjee et al., 2010; Tayh et al., 2019). This situation clarifies that ESBL-negative *Enterobacteriaceae* carries integrons with different resistance genes.

## Conclusion

ESBL-producing-type *Enterobacteriaceae* are the joint causative agent of UTI, especially *E. coli*. Most isolates under study had class I and class II integrons genes reported for the first time as academic work in our locality. Different types of integrons are worrying for clinicians and healthcare safety staff. Therefore, regional and local molecular-level assessments of ESBLs are critical for enhanced management of empiric therapy, especially for patients with repeated UTIs. One of the difficulties in this study was obtaining quality control strains that required 6 months till arrival and choosing the most appropriate history from selected patients.

## Supplemental Information

10.7717/peerj.15429/supp-1 Supplemental Information 1 Raw data.

## Additional Information and Declarations

### Data Availability

The following information was supplied regarding data availability: The sequences are available at GenBank: ON745429, ON745430, ON745431, and ON745432. The raw data is available in the Supplemental Files.
